# Hearing Loss, Loud and Clear: Combined Effect of Noise and Toluene in Workers

**Published:** 2006-08

**Authors:** Ron Chepesiuk

Animal studies have clearly shown that simultaneous exposure to noise and toluene, a clear organic solvent widely used in various manufacturing industries, causes hearing loss. Studies of this interaction in the workplace have been limited, however, and their results inconclusive. Research now establishes, for the first time, a strong correlation between hearing loss in workers and their simultaneous exposure to noise and toluene **[*EHP* 114:1283–1286; Chang et al.]**.

Conducted in a Taiwan adhesive factory, the study included three male study groups: 58 workers exposed only to noise (an average of 85 A-weighted decibels), 58 workers exposed to both toluene and noise, and 58 administrative workers. Air samples were collected from the working areas of the three groups, and sound pressure level meters were used to assess noise levels in the same areas. The researchers also calculated the time-weighted average of noise levels for each group.

The researchers collected data through interviews and physical examinations of the participants, including information on lifestyle and sociodemographic variables such as age, whether respondents smoked or drank, and use of hearing protection. They also administered hearing tests in a soundproof room. A physician conducted an otopharyngeal exam to screen for otitis and other ear problems.

Toluene exposure appeared to increase the risk of hearing loss by as much as six times when compared to loss related to noise exposure only. The workers with the lowest toluene exposure had only a slightly lower risk of hearing loss when compared with those with higher levels of toluene exposure.

The authors acknowledge that the study had three limitations: the small sample size, the inability to measure exposure to high levels of toluene over a long work history, and the lack of available data for estimating hearing loss caused by exposure to toluene alone. They conclude, however, that their study does prove that workers face a greater risk of hearing loss when simultaneously exposed to toluene and noise compared to exposure to noise alone.

The authors believe the current established workplace standard for toluene of 100 ppm does not, by itself, protect against hearing loss for those workers exposed simultaneously to noise. They suggest that effective intervention is needed to improve the occupational safety of such individuals.

## Figures and Tables

**Figure:**
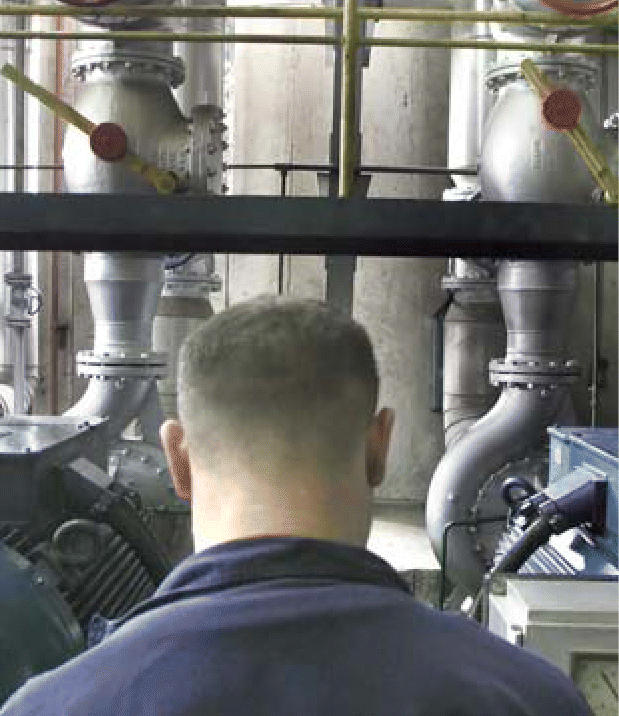
Stereophonic impact? New human data confirm the interactive effect of toluene and noise.

